# Antiplatelet and anti-proliferative action of disintegrin from *Echis multisquamatis* snake venom

**DOI:** 10.3325/cmj.2017.58.118

**Published:** 2017-04

**Authors:** Volodymyr Chernyshenko, Natalia Petruk, Darya Korolova, Ludmila Kasatkina, Olha Gornytska, Tetyana Platonova, Tamara Chernyshenko, Andriy Rebriev, Olena Dzhus, Liudmyla Garmanchuk, Eduard Lugovskoy

**Affiliations:** 1 Protein Structure and Functions Department, Palladin Institute of biochemistry NAS of Ukraine, Kyiv, Ukraine; 2 Educational and Scientific Centre “Institute of Biology” Taras Shevchenko National University, Kyiv, Ukraine

## Abstract

**Aim:**

To purify the platelet aggregation inhibitor from *Echis multisquamatis* snake venom (PAIEM) and characterize its effect on platelet aggregation and HeLa cell proliferation.

**Methods:**

Sodium dodecyl sulfate-polyacrylamide gel electrophoresis (SDS-PAGE) and matrix assisted laser desorption/ionization time-of-flight (MALDI-TOF) were used for PAIEM identification. Platelet aggregation in the presence of PAIEM was studied on aggregometer Solar-AP2110. The changes of shape and granularity of platelets in the presence of PAIEM were studied on flow cytometer COULTER EPICS XL, and degranulation of platelets was estimated using spectrofluorimetry. Indirect enzyme-linked immunosorbent assay was used for the determination of target of PAIEM on platelet surface. An assay based on 3-(4,5-dimethylthiazol-2-yl)-2,5-diphenyltetrazolium bromide was used to evaluate the effect of PAIEM on the proliferation of HeLa cells in cell culture.

**Results:**

The molecular weight of the protein purified from *Echis multisquamatis* venom was 14.9 kDa. Half-maximal inhibitory concentration (IC_50_) of PAIEM needed to inhibit adenosine diphosphate (ADP)-induced platelet aggregation was 7 μM. PAIEM did not affect thrombin- or ADP-induced platelet activation, but it did prevent binding of the anti-IIb antibody to glycoprotein IIb/IIIa (GPIIbIIIa)-receptor of adhered platelets and inhibited the viability of HeLa cells by 54%.

**Conclusion:**

As a member of the disintegrin family, PAIEM inhibited platelet aggregation and cell proliferation possibly by blocking integrin-mediated interactions. However, it did not impair cellular signaling causing any changes in platelet shape and granularity and did not affect ADP-induced platelet degranulation. This disintegrin was shown to be a potent inhibitor of integrin-mediated cellular interactions including platelet aggregation or cancer cell proliferation.

Platelet aggregation represents a multistep adhesion process that is accompanied by fibrin polymerization and leads to thrombus formation (1). This multistep process is triggered by platelet interaction with the subendothelium extracellular matrix containing collagen and von Willebrand factor or by soluble inducers such as adenosine diphosphate (ADP), thrombin or tromboxane A_2_ (2). Platelet aggregation inhibitors used as therapeutic drugs or laboratory reagents mainly target separate distinct stages of the process and can be divided into several groups. Purinergic receptors' antagonists and well-known anti-platelet drugs ticagrelor, clopidogrel, prasugrel, and other similar drugs are used in clinical trials (3) and act mainly on P_2_Y_12_ ADP-receptor (4). Another common anti-platelet agent, aspirin, inhibits thromboxane A2 synthesis in platelets and thus reduces platelet response to agonists (5). There are many novel cyclooxygenase-2 inhibitors that are as potent as acetylsalicylic acid but still have some comparative disadvantages (6). Phosphodiesterase inhibitors decrease the hydrolysis of intracellular cyclic adenosine monophosphate (cAMP) and cyclic guanosine monophosphate (cGMP) and thus attenuate platelet aggregation (7). Some of them are already registered as therapeutic agents, such as cilostazol or dipyridamole.

Peptides and proteins that contain the Arg-Gly-Asp (RGD) sequence are able to inhibit the interaction between platelet glycoprotein IIb/IIIa (GPIIb/IIIa) and fibrinogen, preventing platelet aggregation (8). There are many GPIIb/IIIa antagonists in clinical use, such as abciximab, a Fab-fragment of a monoclonal antibody to GPIIb/IIIa (9), and tirofiban and eptifibatide, heterocyclic organic compounds (10,11).

Disintegrins that contain RGD or Lys-Gly-Asp (KGD) sequences have also been identified in snake venoms (8). Naturally occurring disintegrins could provide a template for the development of synthetic peptide antagonists. Within that context, purified and characterized snake venom disintegrins could be used in clinical trials and studies of platelet aggregation.

The aim of this study was purification, partial characterization, and analysis of anti-platelet effect of the disintegrin from the venom of *Echis multisquamatis* (PAIEM).

## MATERIAL AND METHODS

### Material

Platelet-rich plasma samples were obtained from the blood of healthy donors. Each experiment was replicated using platelets from the blood of three different donors. Volunteers signed informed consent prior to blood sampling according to the Helsinki declaration. Platelet-rich plasma was prepared from human citrated blood by centrifugation at 1000 rpm for 20 min. Washed platelets were obtained from platelet-rich plasma by centrifugation for 15 min at 1500 rpm and re-suspended in 0.004 M HEPES buffer (N-(2-hydroxyethyl)-piperazine-N'-2-ethanesulfonic acid) of рН 7.4 (0.137 М NaCl, 0.003 М КСІ, 0.001 М MgСІ_2_, 0.006 М glucose, 0.003 M NaH_2_PO_4_).

Mouse CD61 monoclonal antibody, goat anti-mouse-horseradish peroxidase conjugated (HRP) antibody, Q-sepharose, Superdex G-75, ABTS (2,2’-azino-di-[3-ethyl-benzothiazoline-6 sulfonic acid] diammonium salt), MTT (3-(4,5-dimethylthiazol-2-yl)-2,5-diphenyltetrazolium bromide, HEPES buffer, and Amicon Ultra-0.5 mL Centrifugal Filters (UFC500324, Merck Millipore) were purchased from Sigma-Aldrich (St. Louis, MO, USA). ADP was purchased from Tekhnologia-standard (Russia).

### Methods

#### Size-exclusion chromatography

The crude venom of *Echis multisquamatis* was dissolved in 0.05 M Tris-HCl buffer of pH 7.4 (tris-buffered saline, TBS) and gel-filtered through Superdex G-75 column. The column volume was 60 ml, with a flow rate of 1 ml/min. Fractions that exhibited anti-aggregatory activity were collected and prepared for the ion-exchange chromatography.

#### Ion-exchange chromatography

Q-sepharose column was pre-equilibrated with 0.05 M Tris-HCl buffer of pH 8.9. The column volume was 3 ml, with a flow rate of 1 ml/min. The solution was eluted with a step gradient of NaCl, and the fraction that contained low-molecular weight fraction was eluted at the ionic strength of 1 M. The active fraction was further concentrated and desalted using Amicon Ultra-0.5 mL Centrifugal Filters (UFC500324, Merck Millipore).

#### SDS-PAGE and mass-spectrometry

The molecular weights and purity of PAIEM were determined by sodium dodecyl sulfate-polyacrylamide gel electrophoresis (SDS-PAGE) 15% gel according to Laemmli (12). Matrix assisted laser desorption/ionization time-of-flight (MALDI-TOF) analysis of purified PAIEM was performed using a Voyager-DE (Applied Biosystems, Foster City, CA, USA). Н+-matrix ionization of polypeptides with sinapine acid (Sigma-Aldrich) was used. Results were analyzed by Data Explorer 4.0.0.0 (Applied Biosystems) (13).

#### Flow cytometry

The shape and granularity of resting platelets in platelet-rich plasma after the incubation with PAIEM *vs* TBS were monitored by flow cytometer as described elsewhere (14). The suspension of washed human platelets in 0.004 M HEPES buffer of рН 7.4 was incubated with 0.1 mg/mL of PAIEM or the equal volume of TBS for the control in the presence of 0.01 M CaCl_2_. Platelet activation was induced by 0.125 National Institute of Health units per mL (NIH/mL) of thrombin (15). Parameters of frontal and orthogonal light-dissipation were monitored for the detection of the change of shape and granularity of platelets during their activation at 1, 1.5, 2, 2.5 minutes from activation point.

#### Spectrofluorimetric measurements of platelet degranulation

Platelet activation was registered with pH-sensitive fluorescent dye acridine orange that is accumulated in secretory granules according to their ΔpH and is released after activation. Platelet-rich plasma with or without addition of 10 μg/mL of PAIEM was preincubated at 37°C for 10 minutes, and fluorescence measurements started after the acridine orange application. Changes in fluorescence intensity were measured in a stirred thermostatted cuvette at excitation and emission wavelengths of 490 and 530 nm, respectively, with slit bands 5 nm each. The ADP (2 µM) was applied at the steady state level of acridine orange fluorescence. The traces were normalized (F_t_/F_0_) to similar data in the absence of platelets. The exocytotic release was calculated as percentage of total accumulated dye at the steady state conditions.

#### Indirect ELISA

Indirect enzyme-linked immunosorbent assay (ELISA) was used for the analysis of PAIEM interactions with activated platelets. Washed human platelets in 0.004 M HEPES buffer of рН 7.4 were added to the wells of 96-well tissue culture plate (Nunc) and then activated by 30 µM of ADP. Wells were gently washed with HEPES buffer trice. Antibody CD61 (specific to IIb subunit of IIb/IIIa platelet receptor) was added to the first row of wells, to the second row antibody CD61 was added alongside with PAIEM (0.05 mg/mL). Probes were incubated for 1 hour at +37°C, then wells were gently washed thrice with HEPES buffer and secondary antibody goat anti-mouse-HRP was added to all wells. After 1-hour incubation, the activity of HRP was monitored using ABTS (2,2’-azino-di-[3-ethyl-benzothiazoline-6 sulfonic acid] diammonium salt). The optical density of the end product was measured at 416 nm. The competition between PAIEM and CD61 antibody was recorded as the difference in signal received in the presence or absence of PAIEM.

#### Cell culture

The HeLa cells (cervical cancer cells) and mouse aortic endothelial cells (MAEC) were grown in Iscove’s modified Dulbecco’s medium (Sigma) supplemented with 10% fetal bovine serum (FBS) and gentamicin (50 μg/mL). All cells were incubated at 37°C in 5% CO_2_. The medium was replaced the next day, and cells were incubated under the same conditions in Iscove’s medium containing 10% FBS, gentamicin (50 μg/mL), and 6 µg/mL PAIEM for 48 hours.

#### Cell viability assay

Cell viability was measured by MTT assay. Cells were plated at 15000 cells per well in 96-well plates and incubated with 100 μL complete medium containing 1 mg/mL MTT at 37°C for 4 hours followed by solubilization with dimethyl sulfoxide (Sigma). The absorbance at 540 nm was measured with a microplate reader. The proliferation was expressed as percentage of the viable cell number of the control (non-treated cells) and PAIEM-treated cells. Cell proliferation rate was calculated as [(1−OD_experimental group_)/OD_control group_] × 100% (16).

### Statistical analysis

Statistical analysis was performed using Microsoft Excel. All assays were performed in series of three replicates and the data were fitted with standard errors using “Statistica 7”. The results were presented as means ± standard error (SD). The difference between the groups was analyzed by one-way ANOVA. The level of statistical significance was set at *P* < 0.05.

## RESULTS

### Purification of the platelet aggregation inhibitor from *Echis multisquamatis* venom

**Figure 1:**
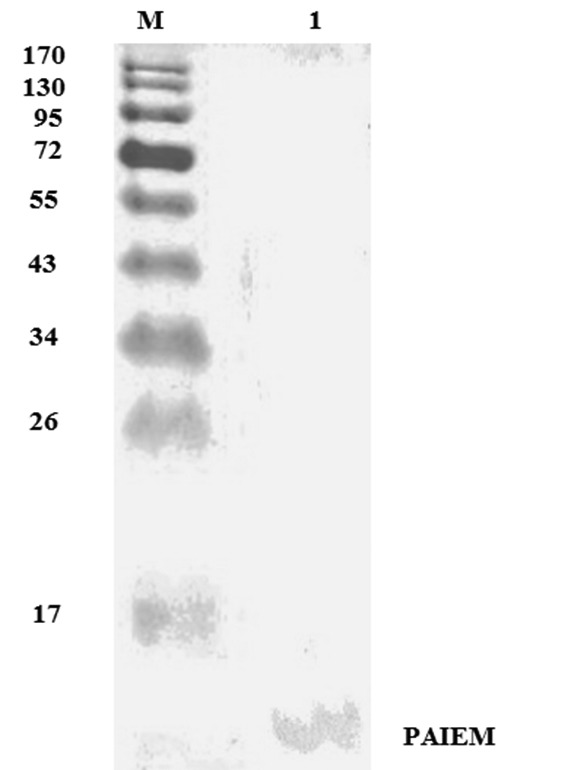
Sodium dodecyl sulfate-polyacrylamide gel electrophoresis **(**SDS-PAGE) of platelet aggregation inhibitor from the venom of *Echis multisquamatis* (PAIEM) eluted from Q-Sepharose and then purified on Superdex G-75 column. M – molecular weight markers.

**Figure 2:**
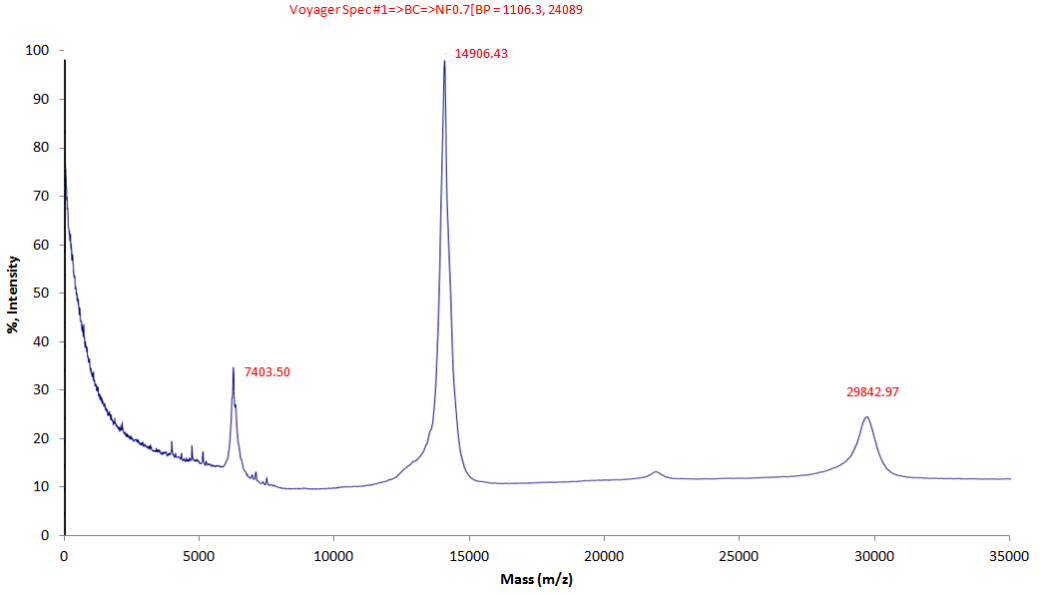
Matrix Assisted Laser Desorption/Ionization Time-of-Flight (MALDI-TOF) analysis of purified platelet aggregation inhibitor from the *Echis multisquamatis* (PAIEM) snake venom. According to MALDI-TOF spectrometry, the protein has molecular weight 14.9 kDa. Other two peaks marked on the graph correspond to 1/2 and 2 charged molecules.

After the crude venom was separated into several fractions by ion-exchange chromatography on Q-Sepharose and the fraction containing PAIEM was purified on Superdex G-75, PAIEM displayed as a single peptide chain on SDS-PAGE profile (Figure 1), since the mobility was the same in the presence and absence of 2% beta-mercaptoethanol. MALDI-TOF-MS analysis showed a molecular mass of 14,9 kDa (Figure 2).

### PAIEM effect on platelets

#### The inhibitory action of PAIEM

**Figure 3:**
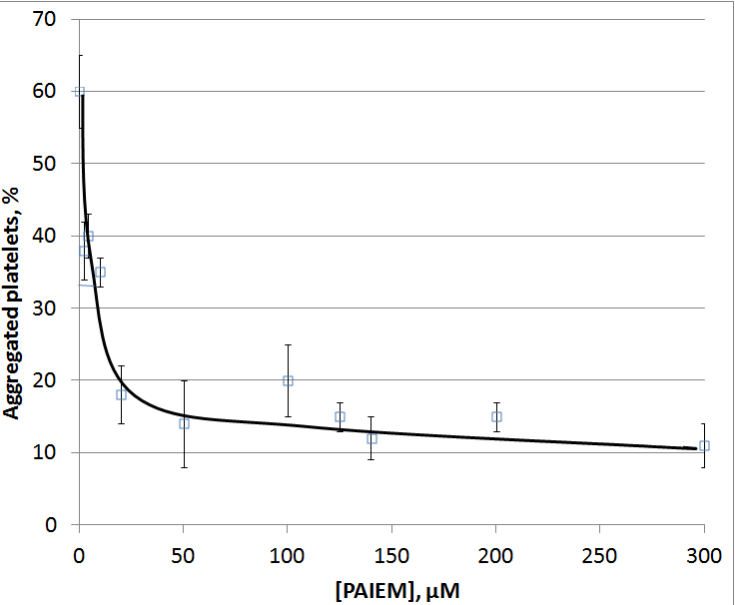
Dose-dependence curve of the level of adenosine diphosphate-induced (2.5 µM) aggregation of human platelets in platelet rich plasma in the presence of platelet aggregation inhibitor from the *Echis multisquamatis* (PAIEM) snake venom. Boxes and whiskers represent the mean values ± standard error, respectively (data of 3 typical experiments made in triplicate). *P < 0.05 as compared to the control.

PAIEM displayed the inhibitory action on platelet aggregation in platelet-rich plasma in dose-dependent manner (Figure 3). The PAIEM IC_50_ on ADP-induced platelet aggregation was 10 µg/mL (approximately 7 μM), and maximum inhibition (about 80%) was shown at 50 µg/mL (Figure 3). However, total inhibition could not be attained by raising PAIEM concentration (up to 300 µg/mL).

**Figure 4:**
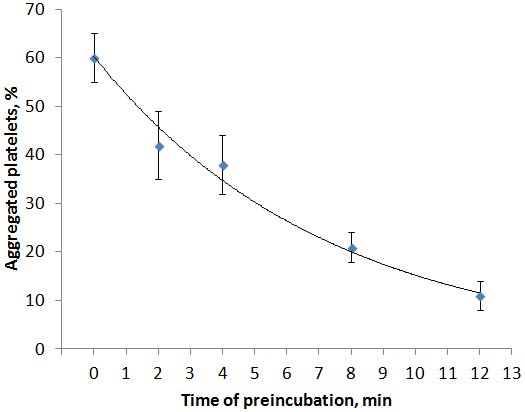
The effect of preincubation of platelet-rich plasma with 5 μg/mL of platelet aggregation inhibitor from the *Echis multisquamatis* (PAIEM) snake venom for 2, 4, 6, 8 12 minutes on the aggregation of platelets induced by 2.5 μM of adenosine diphospate. Data represent the mean values ± standard error (data of 3 typical experiments made in triplicate), *P < 0.05 as compared to the control.

The longer the incubation time of platelet-rich plasma with PAIEM, the greater was the inhibitory effect of the PAIEM on ADP-induced platelet aggregation (Figure 4). Its inhibitory effect on platelet aggregation reached 90% when the incubation time was prolonged to 12 minutes.

#### Platelet activation in the presence of PAIEM

After showing the inhibitory action of PAIEM on platelet aggregation, it was mandatory to know whether PAIEM inhibits activation of platelets. To achieve this goal, we studied thrombin-induced activation of washed platelets in the presence of PAIEM. Dimethyl sulfoxide (DMSO) of 1% was chosen as the positive control. It presumably prevents platelet activation as an inhibitor of cyclooxygenase-1 (17).

**Figure 5:**
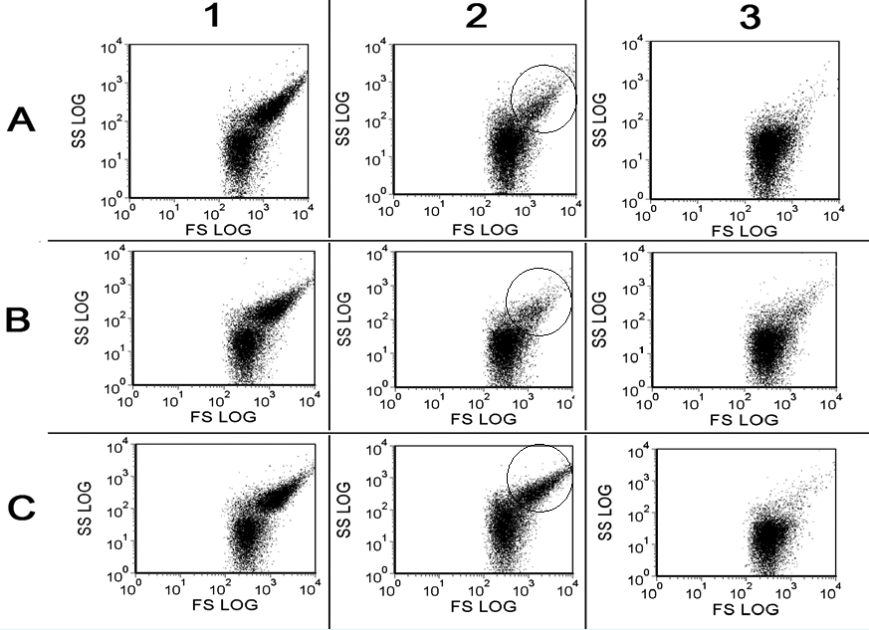
Flow cytometry of washed human platelets activated by 0.125 NIH/mL of thrombin. **1-3** – 1, 2, and 3 minutes after the stimulation, respectively. A. – control probe; B. – in the presence of 50 μg/mL of platelet aggregation inhibitor from the *Echis multisquamatis* (PAIEM) snake venom; C. – in the presence of 1% dimethyl sulfoxide as the inhibitor of platelet activation. SS – side light scattering, parameter of platelets granulation: FS – frontal light scattering, parameter of platelets shape. Traces are typical for 3 independent experiments made in triplicate.

Platelets without blood components were incubated with 50 μg/mL of PAIEM or equivalent volume of DMSO and then activated by 0.125 NIH/mL thrombin. The activation was recorded by flow cytometry, which allows estimating the size of the cells (the change of front light scattering) and the granularity of their cytoplasm (the degree of lateral light scattering) (18). Since the activation of platelets is accompanied by changes in their shape and granulation, the change of front and lateral light scattering showed the level of platelet activation (Figure 5).

Two minutes after the stimulation, thrombin reduced the quantity of platelets in zone of resting cells to 18 ± 5% in control probe (Figure 5, panel A2) and to 16 ± 8% in probe with PAIEM (Figure 5, panel A2). Meanwhile, DMSO distinctly inhibited platelet activation – the level of resting platelets in probe with 1% DMSO was 32 ± 6% (Figure 5, panel C2).

Thus, we assumed that PAIEM does not affect platelet response to thrombin stimulation. We also tested platelet degranulation in the presence of PAIEM by applying the spectrofluorimetric registration of release of granular constituents using pH-sensitive fluorescent dye acridine orange (19).

**Figure 6:**
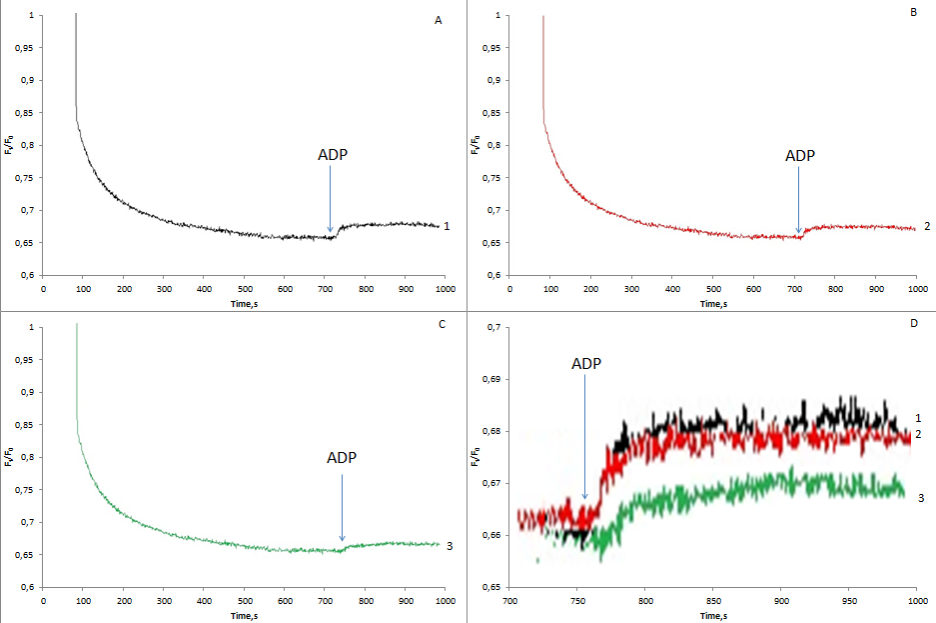
The acidification of platelets secretory granules and the release of granule constituents during adenosine diphosphate (ADP)-induced activation in the presence of platelet aggregation inhibitor from the *Echis multisquamatis* (PAIEM) snake venom. Platelets were loaded with pH-sensitive fluorescent dye acridine orange and stimulated with 2 μM of ADP in the presence or absence of PAIEM. Traces are typical for 5 independent experiments. A – after the addition of equivalent amount of buffer; B – in the presence of 10 μg/mL of PAIEM; C – in the presence of 1% dimethyl sulfoxide; D – summarized enlarged fragment of graphics A-C. Traces are typical for 3 independent experiments performed 5 times each.

The effect of PAIEM *per se* on the acidification of platelet secretory granules was estimated at the steady state conditions of pH-sensitive dye accumulation. The application of PAIEM (50 μg/mL) or TBS in equal volume did not affect the proton gradient of platelet secretory granules. Then platelets were stimulated by ADP (2.5 μM) to detect the release of secretory granules constituents. The degranulation was estimated as percentage of dye release from the total accumulated acridine orange. The comparative analysis showed that ADP-stimulated platelet degranulation was not affected by PAIEM (Figure 6). Thus, we assumed that PAIEM inhibits platelet aggregation and does not affect platelet degranulation or agonist-induced reactivity.

#### Effect of PAIEM on anti-IIb binding to activated platelets

The principal platelet receptor, providing formation and stabilization of fibrin-platelet thrombus, is GPІІb/ІІІа. The activation of platelets triggers a conformational change in the receptor GPIIb/IIIa that leads to the formation of a high-affinity ligand-binding state (20). Multiple binding of fibrinogen molecule to GPIIb/IIIa receptors is a crucial process in platelet aggregation.

**Figure 7:**
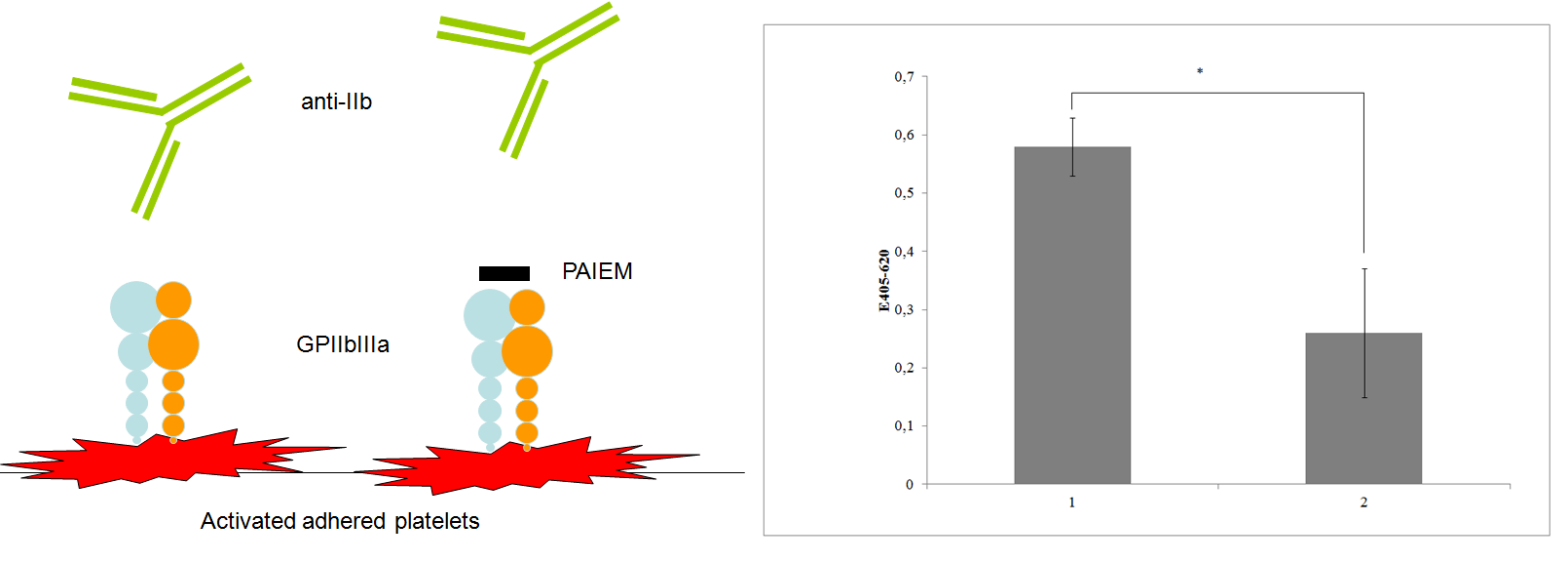
**A.** Scheme of experiment that confirmed the platelet aggregation inhibitor from the *Echis multisquamatis* (PAIEM) snake venom binding to glycoprotein (GP)IIb/IIIa. Washed human platelets were added to the wells of 96-well tissue culture plate and then activated by 30 µM of adenosine diphosphate. Antibody CD61 (specific to IIb subunit of Iib/IIIa platelet receptor) was added to the first row of wells, antibody CD61 was added to the second row alongside with PAIEM (0.05 mg/mL). **B.** Enzyme-linked immunosorbent assay of anti-GPIIb binding to activated platelets in the presence of PAIEM. Boxes and whiskers represent the mean values ± standard error (data of 3 typical experiments performed 5 times each), *P < 0.05 as compared to the control.

Therefore the possible interaction of PAIEM and GPIIb/IIIa was analyzed. For this purpose activated platelets were adsorbed on the surface. The binding of anti-GPIIb to GPIIb/IIIa receptors of adhered platelets was estimated in the presence or absence of PAIEM by ELISA using labeled goat anti-mouse antibody. We showed that PAIEM significantly inhibited the binding of anti-GPІІb antibodies to activated platelets (Figure 7A, B) and assumed that PAIEM prevented platelet aggregation via interaction with GPIIbIIIa and so was probably an GPIIb/IIIa antagonist.

### Effect of PAIEM on proliferation of HeLa and MAIEC cells in cell culture

After showing that PAIEM was a classical disintegrin that inhibited platelet aggregation by binding to GPIIb/IIIa integrins of platelets, we tested the action of PAIEM on proliferation activity on HeLa and MAIEC cells. PAIEM was used at 6 µg/mL that is near IC_50_ for ADP-induced platelet aggregation. Proliferation index was analyzed as the main parameter of cell division and functionality.

**Figure 8:**
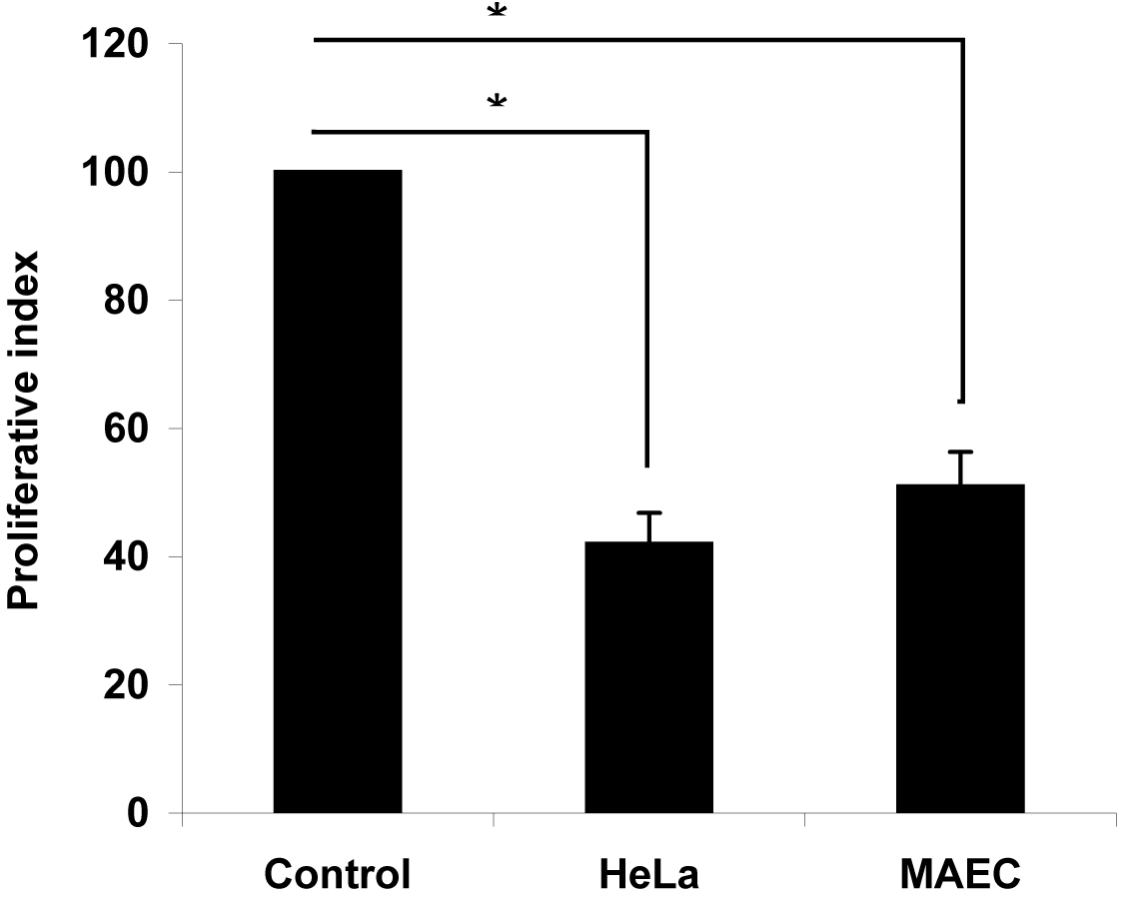
Proliferative index of HeLa and mouse aortic endothelial cells (MAEC) cells in the presence of 6 µg/mL of the platelet aggregation inhibitor from the *Echis multisquamatis* (PAIEM) snake venom *vs* equivalent volume of tris-buffered saline (Control). Cell proliferation rate was calculated as [(1−OD_experimental group_)/OD_control group_] × 100%. The absorbance was measured at 540 nm. Boxes and whiskers represent the mean values ± standard error, *P < 0.05 as compared to the control.

Cell viability after treatment with 6 µg/mL of PAIEM was evaluated by the MTT assay. After 48 hours of incubation, addition of PAIEM decreased the proliferative index by 54% compared to the control group (Figure 8).

## DISCUSSION

In the present study, we isolated and purified a novel disintegrin from *Echis multisquamatis* snake venom by two-step chromatography to homogeneity confirmed by SDS-PAGE and determined that the molecular weight of the PAIEM was 14.9 kDa. We also characterized the PAIEM inhibitory activity on human platelets, finding that PAIEM dose-dependently inhibited ADP-induced platelet aggregation with IC_50_ of 10 µg/mL (approximately 7 μM). Typically, the IC_50_ values of disintegrins vary from 30 to 300 nM. However, in our study, PAIEM exerted its action without the necessary preincubation with platelet-rich plasma, although its inhibitory effect could be slightly increased by incubation up to 12 minutes.

Disintegrins are antagonists of integrin receptors that inhibit their interaction with individual ligands, and in this way play important role in many biological processes including platelet aggregation, inflammation, atherosclerosis and atherothrombosis. Disintegrins received an attention for their potential clinical application to some diseases including thrombosis or cancer. The main function of disintegrins is their inhibitory action on platelet aggregation. Snake venom disintegrins were shown to be potent anti-proliferative agents that prevent cancer cells adhesion (21,22). Some of them were successfully tested as anti-tumor drugs in animal models (23).

It is well known that fibrinogen binds to the exposed integrin GPIIb/IIIa on the platelet surface and mediates platelet interaction and aggregation (24). Disintegrin proved to interact with integrin GPIIb/IIIa and act as fibrinogen receptor antagonist. Thus, disintegrins can inhibit platelet aggregation stimulated by several agonists including ADP, thrombin, and collagen. For example, the disintegrin contortrostatin significantly prevents reocclusion in the canine carotid arterial thrombosis model (25,26). There are several reports on disintegrins from the venom of *Echis multisquamatis.* Previously, a C-lectin type antagonist of collagen GPIa/IIa receptor (EMS16) with molecular weight of approximately 33 kDa was purified and characterized (27-29). Protein with the same molecular weight was reported as fibrinogenase with platelet-modulating activity resulted from cleavage of N-terminal portions of Bβ-chain of fibrinogen (30).

Another low-molecular weight protein with anti-aggregatory activity was purified from the *Echis multisquamatis* venom. Authors determined the molecular weight of the protein as 5.7 kDa (31). It was characterized as anti-GPIIb/IIIa RGD-containing peptide with IC_50_ determined as 97 nM (32).

To summarize results, the low-molecular weight protein from *Echis multisquamatis* venom was shown to be an inhibitor of platelet aggregation. Isolated inhibitor effectively suppresses platelet aggregation in platelet-rich plasma. It did not affect platelet activation or degranulation but prevented platelet aggregation by inhibiting of fibrinogen binding to GPIIb/IIIa receptor. We also showed a pronounced inhibition of HeLa cells proliferation by PAIEM. We can assume that PAIEM inhibited the viability of HeLa cells possibly by blocking their integrin interactions. We can also assume that PAIEM could be a potential platform for the development of anti-thrombotic drugs as well as anti-proliferative agents in anti-cancer therapy. Further studies of PAIEM action on cancer cells will help us to evaluate the possible use of this polypeptide in anti-cancer therapy.
